# Strengthening Multidrug-Resistant Tuberculosis Epidemiological Surveillance in Rio de Janeiro: a multidimensional analysis

**DOI:** 10.1590/0037-8682-0629-2023

**Published:** 2024-07-29

**Authors:** Marcela Bhering, Afrânio Kritski

**Affiliations:** 1 Fundação Oswaldo Cruz, Escola Nacional de Saúde Pública Sergio Arouca, Rio de Janeiro, RJ, Brasil.; 2 Universidade Federal do Rio de Janeiro, Faculdade de Medicina, Programa Acadêmico de Tuberculose, Rio de Janeiro, RJ, Brasil.

**Keywords:** Multidrug-Resistant Tuberculosis, Epidemiological Surveillance, Health Information System, Qualitative Research

## Abstract

This study aimed to reinforce the importance of the epidemiological surveillance of multidrug-resistant tuberculosis (MDR-TB) in Rio de Janeiro State (RJ). Here, we reviewed seven articles we published between 2018 and 2022. This study had two phases. The quantitative phase where frequency was used to describe patient characteristics and regressions were used to evaluate the relationship between treatment outcomes and covariates. The qualitative phase where content analysis of the narratives was performed. Secondary (electronic systems) and primary (semi-structured interviews) data were used. We analyzed 2,269 MDR-TB, 58.1% MDR-TB, and 18.6% extensively drug-resistant TB (XDR-TB) cases, of which 44.3% exhibited unfavorable outcomes. Among the 140 patients with XDR-TB, 29.3% had not undergone prior treatment for MDR-TB. The primary resistance rate in MDR-TB cases was 14.7%, revealing significant demographic and clinical disparities, particularly among women, Caucasians, and those with higher education levels. The number of cases increased from 7.69% in 2000 to 38.42% in 2018, showing an increasing trend (AAPC = 9.4; 95% CI 1.4−18.0, p < 0.001), with 25.4% underreporting. A qualitative study confirmed a high proportion of primary resistance (64.5%) and delayed diagnosis of MDR-TB. In RJ, the diagnostic and therapeutic cascade of MDR-TB must be improved using molecular tests to achieve an early diagnosis of resistance and immediate initiation of appropriate treatment, promote social protection for MDR/XDR-TB patients and their families, enhance TB contact tracing, establish and monitor hospital surveillance centers integrated with Primary Care, and unify various information systems through interoperability for better integration.

## INTRODUCTION

Brazil ranks 14th globally in tuberculosis (TB) cases and 19th in TB-HIV coinfections, representing 0.9% of the estimated global cases and 33% of the estimated cases in the Americas. It is one of the 30 countries prioritized by the WHO for the global elimination of the disease^1^ ^,^ ^2^.

In 2019 and 2023, Brazil reported 73,864 and 80,012 new TB cases, respectively. Despite a decrease in reported cases during the COVID-19 pandemic, there has been an increasing trend in incidence since 2016, rising from 34.3 cases per 100,000 inhabitants in 2015 to 37.0 cases in 2023^3^ ^,^ ^4^.

The risk of TB varies among Brazilian states, ranging from 13.7/100,000 inhabitants in Tocantins to 81.6/100,000 inhabitants in Amazonas^4^. In 2023, the Brazilian capitals reported 29,465 new cases of TB, accounting for 37.1% of the national total. Cities such as Manaus, Rio de Janeiro, Belém, Recife, and Boa Vista stood out with incidence rates exceeding the national average by more than 100%^4^.

Retreatment cases constitute approximately 16.2% of reported cases, with the highest proportions in the Southern (18.8%) and Southeastern (16.3%) regions^5^. In 2023, the Brazilian capitals recorded 7,856 retreatment cases, with the highest percentages in São Paulo, Rio de Janeiro, Manaus and Pernambuco^4^.

Among the new TB cases diagnosed in 2023, 9.3% were HIV coinfection^4^. However, data from 2015 showed an 18.1% coinfection rate among retreatment cases, which is more than double the percentage among new cases^6^. The proportion of new TB cases tested for HIV increased from 68.9% in 2015 to 82.3% in 2023^4^ ^,^ ^6^. In 2023, only 49.8% of these patients received will antiretroviral treatment (ART) during TB treatment^4^.

Regarding deaths, there was a 21.4% reduction from 2004 to 2020, from 2.8/100,000 to 2.2/100,000. In 2019, Brazil recorded 4,532 deaths, of which TB was the underlying cause^4^ ^,^ ^7^. The downward trend observed in the tuberculosis mortality rate in Brazil was consistent with the global temporal pattern. Between 2000 and 2015, mortality decreased by 29% in HIV-negative individuals and 44% in HIV-positive individuals^8^. This reduction can be attributed to a combination of factors, including improved access to diagnosis and treatment, implementation of tuberculosis control strategies, and increased coverage of antiretroviral treatment for people living with HIV^8^. However, the reduction falls short of the 90% reduction target by 2035 set in the End TB Strategy^9^. In 2022, Rio de Janeiro (4.7 per 100,000 inh), Mato Grosso do Sul (3.9 per 100,000 inh), and Amazonas (5.1 per 100,00 inh) had the highest mortality coefficients among states, while Belém (9 per 100,000 inh), Recife (6 per 100,000 inh), São Luis (6.5 per 100,000 inh), and Manaus (5.9 per 100,000 inh) had the highest coefficients among capitals^4^.

With the COVID-19 pandemic, TB deaths are expected to increase in 2021, reaching 5,000, the highest number in the past decade. Of these, 3,600 had TB as an associated cause, with 1,700 cases occurring in people living with HIV/AIDS (PLWHA), representing 63.3% of cases. TB remains the leading cause of death in PLWHA^10^. 

There has also been an increase in the number of new TB cases among vulnerable populations, increasing from 13.7% in 2015 to 15.1% in 2022, with the highest proportion among incarcerated individuals and homeless people^10^. 

This situation is further exacerbated in cases of rifampicin-resistant TB (TB-RR) and multidrug-resistant TB (MDR-TB), which are resistant to rifampicin and isoniazid. Extensively drug-resistant TB (XDR-TB) is resistant to fluoroquinolones and at least one second-line injectable drug (amikacin, kanamycin, or capreomycin), whereas pre-XDR-TB is resistant to either fluoroquinolones or second-line injectable drugs but not both. In 2021, the definitions of pre-XDR and XDR-TB were revised to TB resistant to rifampicin and any fluoroquinolone, and TB resistant to rifampicin plus any fluoroquinolone and at least one of either bedaquiline or linezolid, respectively^11^. Between 2019 and 2020, Brazil reported 3,848 cases of TB resistant to at least one drug, with 66.1% being MDR-TB, 25.8% monoresistant, 7.5% polyresistant, and 0.7% XDR-TB. Rio de Janeiro accounted for the majority of drug-resistant TB cases (23.4%), followed by São Paulo (16.8%), and Rio Grande do Sul (9.5%). Among the new cases of drug-resistant TB, 52.4% achieved successful therapeutic outcomes, 28% were lost to follow-up, 5.7% had failed treatment, and 5.6% died. Rio de Janeiro had the same percentage of therapeutic success but a higher percentage of loss to follow-up (26.2%) and death^12^. 

Despite being one of the most economically developed states in Brazil, Rio de Janeiro stands out for its poor TB control performance, with the second-highest mortality coefficient (4.7 per 100,000 inhabitants in 2022) and third-highest incidence (70.7 per 100,000 inhabitants in 2023), surpassed only by Amazonas^4^. 

This study aimed to reinforce the importance of epidemiological surveillance (ES) for TB, specifically MDR-TB. It seeks to understand the functioning of ES systems based on epidemiological data, clinical-laboratory interfaces, experiences of healthcare professionals treating patients with MDR-TB, and perceptions of the patients themselves. The goal was to contribute to the adoption of public policies that promote better disease control in the Rio de Janeiro (RJ) population.

## METHODS

In this study, the authors reviewed seven articles authored by them and published between 2018 and 2022, addressing MDR-TB in RJ. The study comprised two stages: the first focused on quantitative studies, and the second on qualitative research. Secondary (electronic systems) and primary (interview) data were used.

The quantitative aspect involved retrospective studies with cohorts of MDR-TB patients extracted from the Special Tuberculosis Treatment System (Sistema de Tratamentos Especiais de Tuberculose, SITE-TB): MDR/XDR-TB, HIV-positive/negative/unknown, and primary/acquired resistance. Treatment outcomes were classified as a) cured (the patient should have at least three negative cultures after the 12th month of treatment); b) treatment completed (patients who completed the time stipulated for treatment, with favorable clinical and radiological evolution, but without the cultures of follow-up); c) lost to follow-up (treatment interrupted for 2 consecutive months or more); d) death (death for any reason during treatment); e) failure (Two or more positive cultures out of three recommended after the 12th month of treatment, or three consecutive positive cultures after the 12th month of treatment, at least 30 days apart. Additionally, failure could be reached by medical evaluation and decision to change treatment early due to clinical and radiological worsening; f) unsuccessful (the sum of patients who had the outcome classified as death, failure, or loss to follow-up). Bivariate and multivariate logistic regression analyses were conducted for each outcome. Variables with significance levels < 0.20 in univariate analysis were included in multivariate regression models. Statistical analyses were performed using STATA, version 13.1.

The joinpoint regression technique was applied to analyze the time series of MDR-TB cases. The annual proportion of MDR-TB cases with primary resistance was calculated using the number of MDR-TB cases with primary resistance reported in the year as the numerator, and the total reported MDR-TB cases in the year as the denominator.

To assess the proportion of underreporting of MDR-TB in RJ and identify the associated factors, a probabilistic analysis was conducted. The objective of this study was to identify MDR-TB cases with confirmed results in the Laboratory Environment Management System (Gerenciador de Ambiente Laboratorial, GAL) that were not recorded in SITE-TB and did not receive appropriate TB treatment. The average time (in days) between the examination request and availability of results was calculated for all cases, specifically for notified and underreported cases. Additionally, the time intervals for different stages of the process were calculated from the date of request to the start of TB treatment.

The second stage encompassed qualitative studies and consisted of two cross-sectional studies with nonprobabilistic sampling using the criterion of theoretical saturation. These studies were conducted using semi-structured interviews with MDR-TB patients and healthcare professionals. The theoretical framework adopted was a categorical content analysis, and theoretical saturation was used as the sample criterion. Data were transcribed and processed with the assistance of NVIVO12® software.

This study was conducted at an outpatient care center (CR), which is a state reference for MDR-TB treatment, severe cases of tuberculosis, and other mycobacteriosis in the state of Rio de Janeiro.

### ● Ethics

This study was approved by the Research Ethics Committee (CEP) of the Clementino Fraga Filho University Hospital/Federal University of Rio de Janeiro (CAAE 10126919.2.0000.5257), opinion number 3,373,280, and the CEP of the Sergio Arouca National School of Public Health/Oswaldo Cruz Foundation (CAAE 10126919.2.3001.5240), a co-participating institution, opinion number 3392346.

## RESULTS

### ● First Stage

In the initial study published in Plos One^13^, it was observed that between 2000 and 2016, 2,477 cases of MDR-TB were reported in Rio de Janeiro, with 208 cases excluded due to incomplete information. Among the 2,269 cases analyzed, 1,264 (55.7%) were cured or completed treatment, including 58.1% of MDR-TB cases and 18.6% of XDR-TB cases. The remaining 1005 cases exhibited unfavorable outcomes; 433 (19.1%) were lost to follow-up, 347 (15.3%) died, and 225 (9.9%) progressed to treatment failure (Table 1).

**TABLE 1:** Treatment outcomes among 2269 patients with MDR-TB and XDR-TB.

Categories of drug resistance	Outcomes												
	Cured		Treatment completed		Died		Failed		Defaulted		Total		p-value
	n	%	n	%	n	%	n	%	n	%	n	%	
**MDR-TB**	607	28.6	631	29.6	305	14.3	172	8.1	414	19.5	2129	93.8	<0.001*
**XDR-TB**	15	10.7	11	7.9	42	30.0	53	37.9	19	13.6	140	6.2	
**Total**	**622**	**27.4**	**642**	**28.3**	**347**	**15.3**	**225**	**9.9**	**433**	**19.1**	**2269**	**100**	

MDR-TB: multidrug-resistant TB; XDR-TB: extensively drug-resistant TB. Fonte: PLoS One. 2019 Nov 20;14(11):e0218299. doi: 10.1371/journal.pone.0218299.

In the logistic regression-adjusted model, characteristics such as age < 40 years, less than 8 years of education, Afro-Brazilian ancestry, and illicit substance use were associated with unfavorable outcomes and treatment abandonment. HIV positivity has been linked to unfavorable outcomes and death. The presence of bilateral disease and a history of previous MDR-TB treatment resulted in nearly twice the probability of unfavorable outcomes, whereas XDR-TB presented a 4.7 times higher probability than MDR-TB. Treatment abandonment was associated with male sex, smoking status, and previous MDR-TB treatment. Drug use doubles the probability of treatment abandonment. The presence of bilateral disease, comorbidities, and XDR TB was associated with twice the risk of mortality. Culture conversion at six months was identified as a protective factor for all outcomes, particularly unfavorable outcomes and death (Table 2).

**TABLE 2:** Multivariate analysis: Predictors of unfavourable outcome, default and death among 2269 patients with MDR-TB and XDR-TB.

Predictors	Unsuccess	p-value	Default	p-value	Death	p-value
**Sex**						
Male			1.42 (1.08-1.87)	0.012		
**≥40 years**						
No	1.32 (1.06-1.66)	0.013	1.74 (1.33-2.26)	<0.001		
**Years of study**						
< 8 years	1.61 (1.25-2.06)	<0.001	1.51 (1.12-2.02)	0.006		
**Afro-Brazilian**						
No	1.33 (1.05-1.67)	0.014	1.46 (1.11-1.92)	0.006		
**Drug use**						
Yes	1.78 (1.15-2.75)	0.009	**2.17 (1.42-3.31)**	**<0.001**		
**Smoking**						
Yes			1.66 (1.06-2.61)	0.026		
**Categories of drug resistance**						
XDR-TB	**4.71 (2.67-8.33)**	**<0.001**	0.42 (0.23-0.78)	0.006	**2.54 (1.36-3.01)**	**<0.001**
**Chest radiography**						
Bilateral	2.2 (1.70-2.91)	<0.001			**2.23 (1.50-3.30)**	**<0.001**
**HIV status**						
Positive	1.60 (1.05-2.43)	0.026			1.74 (1.10-2.74)	0.017
**Comorbidities**						
Yes			0.39 (0.22-0.67)	0.001	2.03 (1.36-3.01)	<0.001
**Six-month culture conversion**						
Yes	**0.17 (0.13-0.22)**	**<0.001**	0.45 (0.34-0.61)		**0.07 (0.04-0.13)**	**<0.001**
**Previous MDR-TB treatment**						
Yes	**2.35 (1.79-3.09)**	**<0.001**	1.91 (1.44-2.53)	<0.001		

**MDR-TB:** multidrug-resistant tuberculosis; **XDR-TB:** extensively drug-resistant tuberculosis; **HIV:** human immunodeficiency virus. **Fonte:** PLoS One. 2019 Nov 20;14(11):e0218299. doi: 10.1371/journal.pone.0218299.

Additionally, among the 140 patients with XDR-TB in this study, 41 (29.3%) had not received previous treatment for MDR-TB. This suggests that the patients never underwent second-line drug treatment. Hence, a plausible hypothesis to explain why 29.3% of these patients were in their first MDR TB treatment was that they were initially infected primarily with XDR TB strains (Table 3).

**TABLE 3:** Demographic and clinical characteristics among 2269 patients with MDR/XDR-TB.

Characteristics	MDR-TB		XDR-TB		p-value
	2129		140		
	n	%	n	%	
Chest radiography (n=2191)					
Cavitation	1652	80.4	120	88.2	0.024
Bilateral	1531	74.6	117	86.0	0.003
Drug Resistance type					
Primary	324	15.2	10	7.1	
Acquired	1805	84.8	130	92.9	0.009
Previous MDR-TB treatment					
No	426	20	41	29.3	<0.001
Yes	1703	80%	99	70.7	

**MDR-TB:** multidrug-resistant tuberculosis; **XDR-TB:** extensively drug-resistant tuberculosis. **Fonte:** PLoS One. 2019 Nov 20;14(11):e0218299. doi: 10.1371/journal.pone.0218299.

The researchers then analyzed cases with primary drug resistance in the study cohort. In an article published in the Pan American Journal of Public Health^14^, the primary drug resistance rate among MDR-TB cases was 14.7%, and unfavorable outcomes were 30.3% in the primary drug resistance group and 46.7% in the acquired drug resistance group. Loss to follow-up was the most frequent unfavorable outcome in both groups, with 12.3% of patients with primary drug resistance and 20.3% of those with acquired drug resistance. Death, another unfavorable outcome, was observed in 10.8% of patients in the primary drug resistance group and 16.1% in the acquired drug resistance group.

Significant differences were identified in demographic and clinical characteristics between the two groups. Proportionally, the primary drug resistance group had a higher percentage of cases among women (46.4% compared to 33.5% in the acquired drug resistance group), Caucasians (47.3% compared to 34%), and those with at least eight years of education (37.7% compared to 27.4%). Patients with primary XDR-TB had 12.2 times more chances of treatment failure than those with MDR-TB, and patients with comorbidities had twice the chance of failure in the primary drug-resistant group. The presence of bilateral disease and less than eight years of education were associated with unsuccessful outcomes in both groups. In the primary drug-resistant group, individuals deprived of liberty had eight times more chances of being lost to follow-up. This could be a consequence of the limited interaction between prison healthcare and health services coordinated by municipal and state authorities, which reduces the likelihood of medication adherence.

Regarding HIV coinfection, an article published in the International Journal of Tuberculosis and Lung Disease^15^ revealed that out of a total of 2,269 cases, 1,999 (88.1%) tested negative for HIV, 156 (6.9%) tested positive, and 114 (5.0%) had unknown HIV status. Unfavorable outcomes were more frequent among HIV-positive patients (52.6%) than among HIV-negative patients (43.7%) or those with an unknown HIV status (43.9%).

The success rate among TB-XDR cases that were HIV-negative/unknown was only 20.8%, whereas, during the same period, there were no instances of therapeutic success among HIV-positive TB-XDR cases. Therapeutic failure was nearly the same between the TB-XDR groups, but was lower among TB-MDR cases that were HIV-positive (2.8%), compared to TB-MDR cases that were HIV-negative (8.5%), while death was more frequent in HIV-positive patients, especially in those with TB-XDR (Table 4).

**TABLE 4:** Treatment outcomes among patients with MDR- and XDR-TB by HIV status (n = 2,155).

Outcomes	HIV Negative					HIV Positive				
	MDR		XDR		p-value	MDR		XDR		p-value
	1877		122					11		
	n	%	n	%		n	%	n	%	
Cure/treatment completed	1102	58.7	24	19.7	<0.001	74	51.0	0	0.0	0.001
Lost to follow up	354	18.9	14	11.5	0.041	35	24.1	3	27.3	0.815
Died	258	13.7	37	30.3	<0.001	32	22.1	4	36.4	0.278
Failed	163	8.7	47	38.5	<0.001	4	2.8	4	36.4	<0.001

**MDR-TB:** multidrug-resistant tuberculosis; **XDR-TB:** extensively drug-resistant tuberculosis; **HIV:** human immunodeficiency virus. **Fonte:** Int J Tuberc Lung Dis. 2021 Apr 1;25(4):292-298. doi: 10.5588/ijtld.20.0887.

In the final multivariate Cox regression model, previous treatment for MDR-TB (HR 1.97, 95% CI 1.22-3.18) and illicit drug use (HR 1.68, 95% CI 1.01-2.78) were associated with a higher risk of unsuccessful treatment outcomes. In contrast, culture conversion at six months (HR 0.48, 95% CI 0.27-0.84) and the use of antiretroviral therapy (ART) (HR 0.51, 95% CI 0.32-0.80) were identified as predictors of a lower risk of treatment failure.

In another study published in RSBMT^16^, researchers analyzed the temporal behavior of primary MDR-TB cases. The joinpoint regression model revealed significant growth between 2000 and 2009 (APC: 11.19; 95% CI 2.3-20.8, p < 0.001), a stationary phase between 2009 and 2013 (APC: −10.38; 95% CI 35.6-24.7, p = 0.5), and increasing growth between 2013 and 2019 (APC: 21.85; 95% CI 11.9-32.7, p < 0.001). Analysis of the total period revealed a significant upward trend in the proportion of MDR-TB cases with primary resistance (AAPC, 9.4; 95% CI 1.4-18.0, p < 0.001).

In the last study of this first phase, a retrospective analysis was conducted using secondary data from two electronic systems: GAL, containing information on resistance tests for at least rifampicin and isoniazid (RH) requested and released between August 2010 and May 2017, and SITE-TB, with notified cases of DR-TB that started treatment between January 2008 and December 2018^17^.

Analyzing the data between the two systems, it was observed that of the 651 MDR-TB cases registered in the GAL, 165 were not reported in the SITE-TB, resulting in an underreporting rate of 25.4%. More alarmingly, 61 (37%) of the underreported patients died.

In regression analysis, requests for laboratory tests by hospitals were significantly associated with underreporting (OR of 2.86 and a 95% Confidence Interval (CI) of 1.72-4.73).

The average time elapsed between the test request and result availability was 113 days. For the reported cases, the average period between test request and TB treatment initiation was even longer, totaling 169 days.

### ● Second Stage

Seventy-two patients were treated for MDR TB at the Germano Gerhardt Research Outpatient Clinic-CRPHF at the study period. Thirty-one patients and six health professionals involved in the treatment and monitoring of MDR-TB cases, directly (doctors, nurses, and social workers) and indirectly (pharmacists and professionals responsible for the laboratory), were included between 2018 and 2019. 

Among the patients, 16 (51.6%) were men, with ages ranging from 18 to 65 years, and an average or median age of 43 years. The average monthly family income ranged from USD 150 to USD 1750, with a median of USD 317. Occupationally, 13 (43.3%) patients were unemployed and 12 (40%) received some social benefits from the government. Nine patients (29%) reported previous cases of active TB among their household contacts. 

Among the interviewed patients, the following primary thematic categories were identified:

- Patient journey in search of a diagnosis: Overall, 16 patients (51.6%) received incorrect DS-TB treatment, 12 (64.5%) had primary drug resistance and four (36.4%) acquired drug resistance. Additionally, 12 patients continued standard TB treatment for more than 6 months before treatment failure was detected.
- Previous history of TB: Among 20 patients (64.5%), primary drug resistance was noted, with 14 (70%) reporting familial or occupational TB history; four being household contacts. Patients with prior household contact with TB did not report any active case findings. Currently, 21 patients (68%) receive weekly injectable drugs at primary care and transition to self-administered weekly medication after the intensive care phase.
- Knowledge and attitudes related to MDR-TB: Thirteen patients (41.9%) recognized TB as an airborne or cough-transmitted disease, while five (16%) perceived MDR-TB treatment as more challenging than DS-TB. Only two studies have acknowledged TB's potential fatalities of TB.

Among the secondary thematic categories, the following stood out: a) symptoms, b) delay in the diagnosis of MDR-TB, c) prolonged use of drug-sensitive TB treatment in patients with drug-resistant TB, d) Primary MDR-TB, e) failure to conduct family contact screening, f) abandonment of previous treatment, g) information regarding diagnosis and treatment, and h) Sources of information^18^.

The most relevant observation was that the majority of healthcare professionals were female and nurses. Most of these professionals had a master's degree or specialization in sanitary pneumonia, with an average work experience of 8 years. The main healthcare challenges include increasing MDR-TB cases owing to primary care deterioration and delayed diagnosis. Factors, such as reduced primary care teams, high staff turnover, and inexperienced professionals, contribute to delayed diagnoses. Despite the availability of Xpert MTB/RIF, patients often start sensitive TB treatment without undergoing drug sensitivity tests, which hinders MDR-TB treatment. Insufficient contact tracing leads to treatment delays and dropouts. Economic vulnerability complicates the treatment, understanding, and accessibility. Professional consensus emphasizes the need for improved patient tracking and social support. The main difficulties identified for treatment implementation are a) social vulnerability of patients, b) reduction of family health teams in the primary care sector, c) difficulties in locating missing patients, and d) lack of integration between the laboratory information system and epidemiological surveillance systems. In contrast, some facilitators were observed, such as: a) adequate biosafety measures in the service environment and b) an adequate number of healthcare professionals in the tertiary reference where the study was conducted^19^.

## DISCUSSION

During the study period, the high proportions of treatment failure (37.9%) and death (30.0%) among MDR-TB cases reflect the limitations of the available therapeutic options and the urgent need for Brazil's healthcare system to incorporate new drugs into MDR-TB treatment. In this scenario, efforts should be directed toward increasing cure rates, reducing loss to follow-up, improving vulnerable populations’ access to healthcare services, expanding social protection measures, and implementing public policies to prevent new cases of drug resistance.

Simultaneously, in the state of Rio de Janeiro, the observed increasing trend from 2000 to 2019 indicated an increase in primary MDR-TB cases from transmission sources, making the elimination of MDR-TB even more challenging. Primary resistant cases of MDR-TB represent a distinct source of transmission, which is linked to other chronic diseases that increase the risk of contracting TB. The unfavorable treatment outcomes observed in the state of Rio de Janeiro may contribute to the increased transmission of primary MDR-TB, further worsening drug resistance.

This underscores the importance of conducting rapid molecular tests, recommended by the World Health Organization, such as Xpert Ultra, Line Probe Assay, Truenat, and Targeted Next-Generation Sequencing (T-NGS), to enhance the diagnosis of primary drug resistance, and emphasizes the urgency of expanding strategies to reduce MDR-TB transmission.

Regarding patients co-infected with HIV in Rio de Janeiro, MDR-TB and HIV treatments were conducted in different health units, and the surveillance systems for both diseases were not interconnected. This makes the monitoring of treatment and adverse events challenging. Owing to the lengthy duration of MDR/XDR-TB treatment (18-24 months) and the large number of pills required for concomitant treatment, close surveillance by healthcare professionals is necessary. The co-administration of drugs in patients using antiretroviral therapy (ART) and MDR/XDR-TB drugs is complex, highlighting the importance of training healthcare professionals to recognize potential additive toxicity due to concurrent regimens.

In our study, a high percentage of underreporting in patients with laboratory diagnoses was observed, as well as a long time between diagnosis and treatment initiation, emphasizing the need for interoperability between electronic systems to provide a more efficient and timely approach in the GAL and SITE-TB systems, thus avoiding the underreporting of MDR-TB and its negative consequences.

Qualitative research confirmed the results of a high proportion of patients with primary resistance (64.5%), delays in MDR-TB diagnosis, low knowledge of TB and MDR-TB among patients, and low adherence to active contact tracing and follow-up in Primary Care. These results suggest that delays in MDR-TB diagnosis led patients to undergo unnecessary treatment, worsen clinical conditions, increase catastrophic costs, and perpetuate the disease transmission chain. Even with a laboratory network capable of diagnosing MDR-TB in Rio de Janeiro, this has not been sufficient to expedite the initiation of correct treatment for MDR-TB.

In conclusion, in Rio de Janeiro State, with low performance in MDR-TB control associated with the increase in primary MDR-TB, there is a urgent need to: a) improve the diagnostic and therapeutic cascade of MDR-TB, using molecular tests as the first approach for a patient with presumed DR-TB to achieve early diagnosis of resistance to first and second-line drugs and new drugs, and immediate initiation of appropriate DR-TB treatment; b) promote social protection for MDR/XDR-TB patients and their families; c) enhance TB contact tracing; d) establish and monitor hospital surveillance centers and TB notification routines in hospitals integrated with Primary Care; e) unify various information systems through interoperability, making them more agile and integrated.
